# Antioxidant Activity and the Potential Mechanism of the Fruit From *Ailanthus altissima* Swingle

**DOI:** 10.3389/fvets.2021.784898

**Published:** 2021-12-13

**Authors:** Ya-nan Mo, Feng Cheng, Zhen Yang, Xiao-fei Shang, Jian-ping Liang, Ruo-feng Shang, Bao-cheng Hao, Xue-hong Wang, Hong-juan Zhang, Ahmidin Wali, Chun-fang Lu, Yu Liu

**Affiliations:** ^1^Key Laboratory of New Animal Drug Project of Gansu Province, Key Laboratory of Veterinary Pharmaceutical Development, Ministry of Agriculture and Rural Affairs, Lanzhou Institute of Husbandry and Pharmaceutical Sciences of Chinese Academy of Agriculture Sciences, Lanzhou, China; ^2^Key Laboratory of Plant Resources and Chemistry in Arid Regions, Xinjiang Technical Institute of Physics and Chemistry, Chinese Academy of Sciences, Urumqi, China; ^3^College of Veterinary Medicine, Gansu Agricultural University, Lanzhou, China

**Keywords:** *Ailanthus altissima* Swingle, antioxidant activity, RAW264.7 cell, network pharmacology, *in vitro*, antioxidant mechanism

## Abstract

The fruits of *Ailanthus altissima* Swingle (AS) possess a variety of pharmacological activities. Its antioxidant activity and the potential mode of action have not yet been investigated. In *in vitro* studies, AS revealed the strong reducing power and DPPH scavenging effect, but hydroxyl radical scavenging activity and ferrous ions-chelating ability were not strong. Meanwhile, the oxidative stress RAW264.7 cell injury model was established, the low and medium-doses of AS showed significant protective effects on the viability of H_2_O_2_-treated cells by CCK-8 method. Besides, three doses of AS all increased the activities of SOD, CAT, and GSH-Px and decreased the MDA level compared with the H_2_O_2_ group, suggesting it significantly relieved oxidative stress of cells. The active ingredients and related targets of AS were collected by HERB and Swiss Target Prediction database, the common targets of drugs and diseases database were conducted by GeneCards database platform and the Venny platform. We screened the core targets of AS like threonine kinase1 (AKT1), mitogen-activated protein kinase 1 (MAPK1), sirtuin-1 (SIRT1), mechanistic target of rapamycin kinase (MTOR) by STRING database, and the key pathways involved PI3K-AKT and FoxO signaling pathway by KEGG pathway enrichment analysis. Besides, qRT-PCR revealed AS preconditioning significantly up-regulated the expression level of AKT1, SIRT1, MAPK1, and MTOR in model cells, and the effect was related to the regulation of FoxO and PI3K/AKT signaling pathway. In summary, AS showed significant antioxidant activity and its potential mechanism was regulating FoxO and PI3K/AKT signaling pathway.

## Introduction

Oxidative stress is the imbalance between oxidation and antioxidant reaction caused by the accumulation of free radicals in the body (1). Cells produce free radicals through multiple metabolic pathways, and free radicals are crucial factors that give rise to oxidative damage of proteins, showing high reactivity (2). Reactive oxygen species (ROS) are the uppermost free radicals in cells and mainly produced by mitochondria (3). Excess ROS break down cells and tissues, affect metabolic function, and cause different health problems (4). In addition, previous studies have indicated that oxidative damage to proteins caused by ROS has something to do with aging, the occurrence of atherosclerosis, arthritis, cancer, and neurodegenerative diseases such as Alzheimer's and Parkinson's disease (5–7). Cells maintain ROS homeostasis through superoxide dismutase (SOD) system, catalase (CAT) system, glutathione peroxidase (GSH-Px) system, etc. (8).

Antioxidants are a class of substances that help trap and neutralize free radicals, thereby they can decrease the damage to the body caused by free radicals. Additionally, antioxidants play a protective role against certain diseases including inflammation and cancer caused by oxidative stress (9, 10). Antioxidants are classified as synthetic antioxidants and natural antioxidants generally. However, considering several synthetic antioxidants have presented toxicity and side effects, especially with long-term use (11–13), the exploration of safe and side-effect-free natural antioxidants has become a hot spot for the past few years. Some natural products, chiefly the extracts of some medicinal plants, have strong antioxidant activity with little toxicity and side effects (14). For this reason, antioxidants from natural sources have a good application prospect in the prevention and treatment of various diseases related to oxidative stress.

*Ailanthus altissima* (Mill.) Swingle (Simaroubaceae), the tree-of-heaven, is an early successional tree, native to China and North Vietnam (15). Its fruit is a traditional Chinese medicine, named “FENG YAN CAO” in Chinese, which has the effect of clearing heat and dryness, stopping dysentery and bleeding, and can also be used to treat diarrhea, heat ailments, epilepsy, trichomonas vaginalis, and ophthalmic disease (16, 17). Previous studies on the composition of AS have revealed the presence of alkaloids, terpenoids, steroids, and flavonoids (18). Jin et al. (19) found a decoction of *Ailanthus altissima* (AS) could inhibit inflammatory cytokines production, such as TNF, IL-6, IL-8, as well as NF-κB. The EtOH extract of AS decreased the generation of the cyclooxygenase-2-dependent phases of prostaglandin D2 in bone marrow–derived mast cells (20). A variety of studies have investigated the anti-inflammatory effects of AS, while little research has been done to reveal the antioxidant effects of AS. Therefore, this paper focused on investigating *in vitro* and intracellular antioxidant activity and the potential mechanism of AS based on network pharmacology.

This experiment investigated antioxidant activities of AS *in vitro* and its protective effect on H_2_O_2_-induced RAW 264.7 cells. Besides, we explored the potential antioxidant mechanism of AS based on network pharmacology analytical methods and verified it to some extent by qRT-PCR, aiming to provide reference for development of AS and other traditional natural products.

## Materials and Methods

### Material Preparation

The fruits of *Ailanthus altissima* Swingle were purchased from Bozhou Baohua Pharmaceutical Co., Ltd., in August 2020. The plant specimen (2020011) was deposited in Key Laboratory of Veterinary Pharmaceutical Development of Ministry of Agriculture, Lanzhou Institute of Husbandry and Pharmaceutical, Chinese Academy of Agricultural Sciences. The AS-extractum was extracted with 95% ethanol by reflux four times, and the extracted solution was combined and concentrated under reduced pressure to remove the ethanol.

### Materials and Reagents

2, 2-diphenyl-1-picrylhydrazyl (DPPH) was purchased from TCI Shanghai, ethylene diamine teraacetic acid (EDTA) and ascorbic acid (VC) were purchased from Sinopharm Chemical Reagent Co. (Shanghai, China); all other chemicals used were analytical grade and bought from local suppliers. Dulbecco's modified Eagle's medium (DMEM) high glucose and fetal bovine serum were purchased from HyClone (Ultah, US) and Gibcol Life Technology (New York). Cell Counting Kit-8 (CCK-8) was purchased from Biosharp Life Sciences (Hefei, China). CAT, SOD, GSH-Px, and MDA assay kits were obtained from Solarbio Science and Technology Co., Ltd. (Beijing, China). Murine macrophage cell line RAW264.7 cells were purchased from the Type Culture Collection of Chinese Academy of Sciences (Shanghai, China). Simply P Total RNA Extraction Kit was purchased from BioFlux (Hangzhou, China). Prime ScriptTM RT reagent Kit with gDNA Eraser and TB Green^®^ Premix Ex Taq™ II were obtained from Takara (Beijing, China).

### Antioxidant Assay *in vitro*

#### Scavenging Activity Against DPPH Free Radical

The DPPH free radical scavenging activity of alcohol extract from AS was measured and modified slightly according to a previous method by Guo et al. (21). The 1 mM DPPH solution dissolved in 75% ethanol was prepared and protected from light prior to measurement. A total of 100 μL of the alcoholic extract of AS in different concentrations was mixed fully with 200 μL DPPH solution separately in 96-well-plates. The final concentration of the extract was 1, 0.5, 0.25, 0.125, 0.0625, 0.03125, and 0.015625 mg/mL, respectively. The mixture was placed in the dark for 30 min, and the absorbance value was measured at 517 nm. VC was used as a positive control. Each sample was repeated three times. The scavenging rate of the DPPH free radical was calculated using the following equation:


$$\begin{array}{l} \text{Scavenging effect}\left(\%\right)\;=\;\left[\text{1}-\left(A_{i}-A_{j}\right)/A_{0}\right]\times 100\% \end{array}$$

where *A*_0_ is the absorbance of the DPPH solution without sample; *A*_*i*_ is the absorbance of the test sample mixed with DPPH; and *A*_*j*_ is the absorbance of the sample without DPPH.

#### Ferrous Ion-Chelating Ability

The chelating capacity of ferrous ions was determined based on the method described by Senevirathne (22) with some modifications. The chelating ability of alcohol extract from AS was determined by using ferrozine. A total of 100 μL of different concentrations of the AS extract was transferred to the 96-well-plate. Then, it was mixed with 5 μL of the FeCl_2_ solution (2 mM), 20 μL of the ferrozine (5 mM), and 75 uL of the distilled water successively. Let stand for 10 min at room temperature. The absorbance value was measured at 560 nm. The ferrous ion-chelating ability was calculated by the equation:


$$\begin{array}{l} \text{The ferrous ion}-\text{chelating ability}\left(\%\right)\;=\;\left[\text{1}-\left(A_{i}-A_{j}\right)/A_{0}\right]\\ \;\times 100\% \end{array}$$

where *A*_*i*_ is the absorbance of the extract sample mixed with the reaction solution. *A*_*j*_ is the absorbance measured with distilled water instead of ferrozine. *A*_0_ is the absorbance measured with distilled water instead of the extract sample.

#### Hydroxyl Radical Scavenging Activity Assay

The method we used was modified based on previous reporting (23). We mixed 50 μL of the alcohol extract solution of AS (0.0625–4 mg/mL) and 50 μL of the 6 mM FeSO_4_ solution together. Then add 50 μL of the 6 mM salicylic acid-ethanol and the same H_2_O_2_ solution into the mixture, respectively. The mixture incubated at 37°C for 30 min. The absorbance of the mixture was measured at 510 nm against a blank. The hydroxyl radical scavenging ability was calculated by the following equation.


$$\begin{array}{l} \text{Scavenging effect}\left(\%\right)\;=\;\left[\text{1}-\left(A_{i}-A_{j}\right)/A_{0}\right]\times 100 \end{array}$$

where *A*_*i*_ is the absorbance of the extract sample mixed with the reaction solution. *A*_*j*_ is the absorbance measured with distilled water instead of H_2_O_2_. *A*_0_ is the absorbance measured with distilled water instead of the extract sample.

#### Ferric-Reducing Power Assay

The ferric reducing power of alcohol extract from AS was determined and minor modifications were made according to the method of Shang (11). A total of 100 μL of the alcoholic extract of AS in different concentrations was mixed fully with 250 μL of sodium phosphate buffer (pH 6.6) and 250 μL of potassium ferricyanide (1%, w/v). There was 250 μL of trichloroacetic acid (10 wt%) added after the mixture has been reacted at 50°C for 20 min. The commixture was centrifuged at 4,000 rpm for 10 min. There was 50 μl of supernatant taken and mixed with 50 μl of distilled water and 50 μl of ferric chloride (0.1 wt%) in the 96-well-plate. Mix it well and let it stand for 10 min. The absorbance was measured at 700 nm. VC was the positive reference reagent. All *in vitro* experiments were performed at least in triplicate.

### Measurement of Effect Against H_2_O_2_-Induced Oxidative Stress

#### Cell Culture

RAW264.7 was incubated in a DMEM high glucose medium supplemented with 10% fetal bovine serum and maintained at a temperature of 37°C within a humidity incubator containing 5% CO_2_.

#### CCK-8 Assay

RAW264.7 cells were seeded in 96-well-plates at a density of 1 × 10^6^ cells/mL in culture medium for 4 h. Cells were exposed to 100 μL of culture medium containing different concentrations of AS (dissolved in sterile water) or H_2_O_2_ and incubated for 24 h. Detection of cell viability stimulated with AS under oxidative stress was performed as follows: cells with a density of 1 × 10^6^ cells/mL were seeded in 96-well-plates and incubated for 4 h. Then they were exposed to fresh DMEM with different concentrations (0, 30, 50, and 70 μg/mL) of AS for 20 h. The positive control group and the AS-treated groups were then treated with H_2_O_2_ (400 μM) for 4 h. After that, the culture medium was removed and washed with phosphate buffered saline (PBS), and then 100 μL CCK-8 solution (100 μL DMEM for 10 μL CCK-8) was added to each well. The absorption values were measured at 450 nm, using a microplate reader (BioTek, USA), after incubation at 37°C for 4 h. The results were indicated as the percentage viability according to the following equation:


$$\begin{array}{l} \text{Viability }\left(\%\right)\;=\;\left(A_{t}-A_{0}\right)/\;\left(A_{c}-A_{0}\right)\times 100\% \end{array}$$

where *A*_*t*_ is the absorbance of the treatment group. *A*_0_ is the absorbance of the blank control group. *A*_*c*_ is the absorbance of the control group.

#### Evaluation of Antioxidant Enzyme Activity and Lipid Peroxidation

RAW264.7 cells were seeded in 6-well-plate at a density of 1 × 10^6^ cells/mL in culture medium for 4 h and stimulated with AS (0, 30, 50, and 70 μg/mL) for 20 h. The positive control group and AS-treated groups were then exposed to H_2_O_2_ (400 μM) for 4 h. The activity of SOD, CAT, GSH-Px, and MDA in cells was determined using a commercial kit according to the manufacturer's instructions.

### Network Pharmacology Analysis

#### Related Gene Targets Database Construction

The active ingredients of AS were manually obtained by searching “FENG YAN CAO” in HERB (Available online: http://herb.ac.cn/). In addition, the screening conditions were limited to drug likeness ≥0.18 in Swiss ADME (available online: http://www.swissadme.ch/). Second, the SMILE structure of known active ingredients was obtained by PubChem database (available online: https://pubchem.ncbi.nlm.nih.gov/), which was imported into Swiss TargetPrediction database (available online: http://www.swisstargetprediction.ch/) to obtain the target gene corresponding to the active ingredient (24).

#### Common Targets of Drugs and Diseases Database Construction

The GeneCards database platform (available online: https://www.genecards.org/) was used to retrieve the keyword “anti-oxidation” to collect target genes associated with oxidation. Then the common target genes of the active component target genes of AS and antioxidant target genes were obtained by the Venny platform (available online: http://bioinfogp.cnb.csic.es/tools/venny/) (25, 26).

#### Construction of Protein-Protein Interaction Network and Screening of Core Targets

The potential targets of AS in the treatment of oxidative stress were imported into the STRING database (available online: https://string-db.org/). Set the conditions “Minimum required interaction score=0.4” and “Hide disconnected nodes in the network” to obtain protein interaction information including the node degree value. PPI network diagram was drawn by Cytoscape_v3.6.0. The core targets were selected according to the node degree value (27, 28).

#### GO Analysis and KEGG Enrichment Analysis of Core Target Gene

In short, computational R-package of “ClusterProfiler version 4.1.0” was applied for the enrichment analysis of gene ontology (GO) in molecular function (MF), cellular component (CC), and biological function/process (BP), and Kyoto Encyclopedia of Genes and Genomes (KEGG) pathway enrichment analysis was performed by Metascape (available online: https://metascape.org/gp/index.html) on the common targets (29). Furthermore, the visualization bubble chart and histogram were formed and displayed.

#### qRT-PCR Verification Assay

RAW264.7 cells were treated with AS (0, 30, 50, and 70 μg/mL) for 20 h as previously described. They were then exposed to H_2_O_2_ (400 μM) for 4 h and the negative control groups were incubated without treatment for 28 h. We used Simply P Total RNA Extraction Kit to extract total RNA. RNA extract was subsequently DNase treated by using Prime Script^TM^ RT reagent Kit with gDNA Eraser following the manufacturer's instructions. Reverse transcription of RNA and the quantitative expression of the genes was performed with TB Green^®^ Premix Ex Taq™ II according to the manufacturer's instructions. In addition, real time quantitative PCR was performed on QuantStudio 6 Flex (ABI, US). The reaction condition was subjected to an initial predegeneration step at 95°C for 30 s, followed by 40 cycles of 95°C for 5 s and 60°C for 34 s and the last 95°C for 15 s, 60°C for 1 min, and 95°C for 15 s. The target genes were amplified with the primers in Table 1, and GAPDH was used as the internal reference gene. The total reaction system was 20 μL, each reaction was repeated three times, and the QuantStudio^TM^ Real-Time PCR software was used for analysis. The expression quantity of the target genes was calculated by the 2^−ΔΔCT^ method.

**Table 1:** Primers used for qRT-PCR.

**Gene symbol**	**NCBI RefSeq no**.	**Sequence (5′→3′)**
GAPDH	NC_000072.7	(F)GGTTGTCTCCTGCGACTTCA
		(R)TGGTCCAGGGTTTCTTACTCC
AKT1	NC_000078.7	(F) ACAGCCTCCCTCCATCACTTCAG
		(R) TACCCACAATCTACCTCCCACCATC
MTOR	NC_000070.7	(F) TCCATTCCGTCAGCAGCATTGTC
		(R) TCAGCCACACTCCTCATCCTCAC
MAPK1	NC_000077.7	(F) GGTATCCAGCACATGATCCACAGTC
		(R) GCAAGCGTTCTACATCAAGTTACATCC
SIRT1	NC_000076.7	(F) GGATGGCCAGACTTTGCAGC
		(R) CACCAGGGTCCTGCATCCAT

### Statistical Analysis

Experimental data was expressed as the mean ± SE of three independent experiments and analyzed by ANOVA using IBM SPSS Statistics 23. *P* < 0.05 were considered statistically significant.

## Results and Discussion

### Antioxidant Assay *In vitro*

#### Scavenging Activity Against DPPH Free Radical

**Figure 1:**
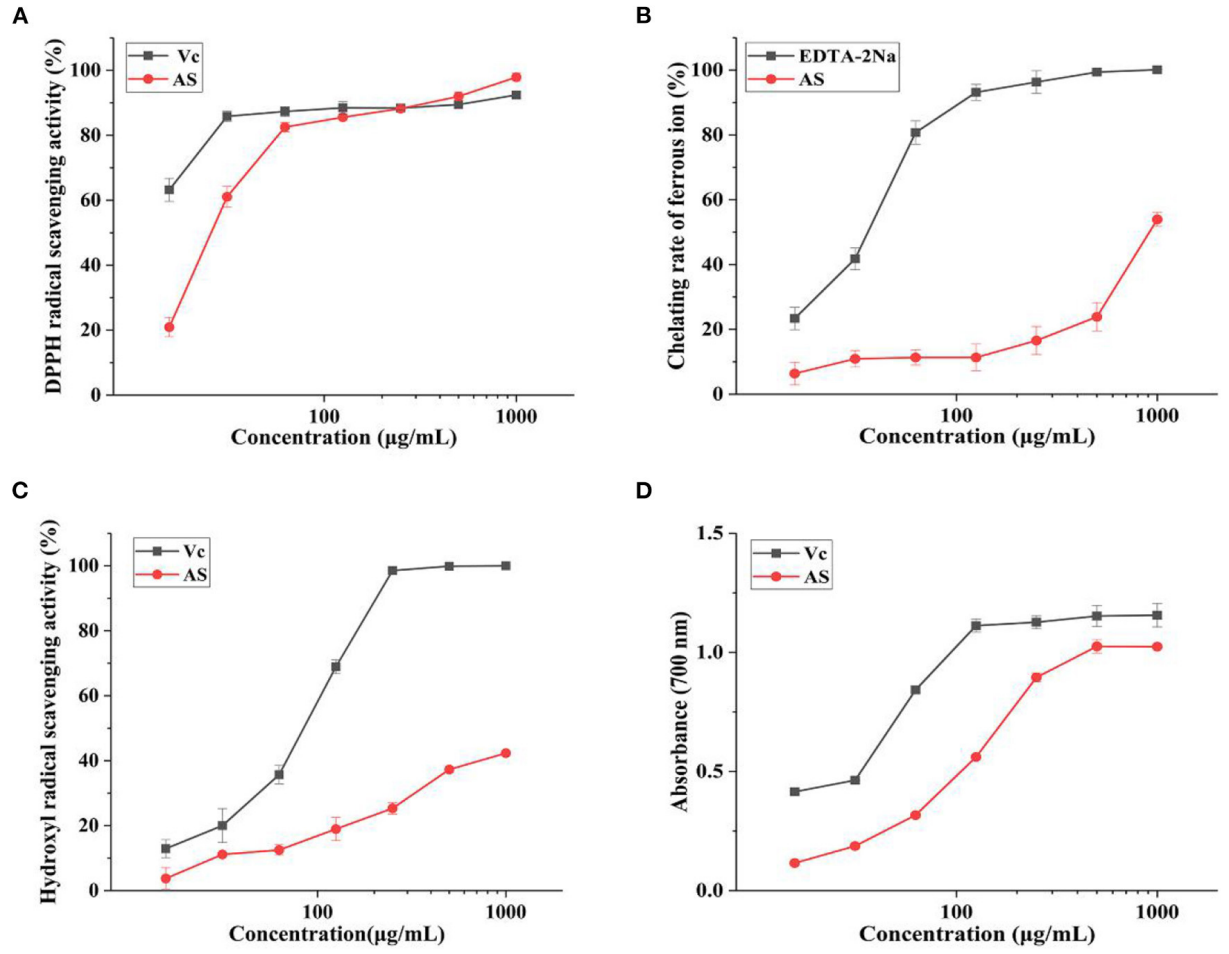
Antioxidant activities of AS at different concentrations **(A)** DPPH radical scavenging activity of AS and VC. **(B)** Ferrous ion-chelating rate of AS and EDTA-2Na. **(C)** Hydroxyl radical scavenging activity of AS and VC. **(D)** Reducing power of AS and VC. Data are shown as the mean ± SD (*n* = 3) (Supplementary Table 4).

DPPH method is one of the most well-known methods for assessing antioxidant activity *in vitro* (30, 31). Figure 1A showed the scavenging activities of AS on the DPPH radical compared with VC. At the lowest concentration (15.625 μg/mL), AS still had a scavenging effect of 20.95% for DPPH. What is more, the scavenging activity was increased with the rise of the concentration of the extract of AS, which could indicate a close-dependent relationship between the scavenging effect and the concentration of AS in the range of 15.625 μg/mL to 1 mg/mL. When the concentrations of AS were 0.5 and 1 mg/mL, the scavenging rates of DPPH free radical were 91.97 and 97.90%, even greater than DPPH radical scavenging activity of VC at the same concentration. VC is recognized as a powerful antioxidant. So, compared with VC, we thought AS presented extremely strong scavenging effects on DPPH free radical.

#### Ferrous Ion-Chelating Ability

Antioxidants are commonly used as metal ion chelators to prevent free radical chain reactions (32). As can be seen from Figure 1B, ferrous ion-chelating ability remarkably increased with the elevation of AS levels from 125 to 1,000 μg/mL. The chelating ability of AS was 53.94% under the concentration of 1,000 μg/mL, so the EC_50_ was roughly speculated to be 1,000 μg/mL in general. While the chelating rate of EDTA-2Na was 41.78 and 80.75% at 31.25 and 62.5 μg/mL, respectively. The EC_50_ of EDTA-2Na was nearly 46.88 μg/mL. As we all know, EDTA-2Na is a strong complexing agent, always used for chelating metal ions and separating metals. Thus, compared with EDTA-2Na, we supposed that AS has certain ferrous ion-chelating ability, but this ability was not strong.

#### Hydroxyl Radical Scavenging Activity Assay

Hydroxyl radical is the most active reactive oxygen species, which can directly react with lipids and their main oxidation products (33). So, the ability of antioxidants to remove existing hydroxyl radicals is important. We found AS had the hydroxyl radical scavenging ability at the concentration between 15.625 and 1,000 μg/mL as shown in Figure 1C. Moreover, the scavenging activity of AS was significantly concentration dependent. The scavenging ability of AS was lower than that of VC. However, VC itself had an extreme scavenging activity against hydroxyl radicals. This result was also represented in the figure. The hydroxyl scavenging rate of AS was 42.34% at the highest concentration (1,000 μg/mL), lower than 50%, while the DPPH scavenging rate of 50% was detected between 15.625 and 31.25 μg/mL. So, the EC_50_ of AS was more than 1,000 μg/mL and the EC_50_ of VC was nearly 23.44 μg/mL. The hydroxyl radicals scavenging capacity of AS was weak in comparison with VC. Therefore, AS had a stronger scavenging capacity for DPPH than hydroxyl radicals. According to previous experiments, the antioxidant mechanism might be due to the supply of hydrogen by antioxidants, which were bound to free radicals and formed stable free radicals to terminate the free radical chain reaction or combine with radical ions (34, 35). Hence, it could conceivably be the hypothesis that AS could be used as electron or hydrogen donors to scavenge radicals.

#### Ferric-Reducing Power Assay

Figure 1D illustrated that within the range of experimental concentration, the reducing power of AS showed a certain positively correlated dose-effect relationship. The curve shape of AS was similar to VC, but the reducing power of the sample was slightly weaker than VC. The absorbance (700 nm) of AS was 1.03, while that of VC was 1.15 under the concentration of 500 μg/mL. This result identified the reducing power of AS was close to VC. Based on the strong reducing power of VC, we concluded AS had a strong reducing power. The reducing power of antioxidants was generally achieved by giving away hydrogen atoms or breaking free radical chains (36). Polyphenols have a structure in which the benzene ring is linked to the hydroxyl group and the hydrogen on the hydroxyl group linked to the benzene ring is unstable and is usually a very good donor of hydrogen or electrons (37). Therefore, AS has a strong reducing ability maybe due to the polyphenols in AS.

Based on the previous findings, we hold the opinion that AS has the strong reducing power and DPPH the scavenging effect. In addition, hydroxyl radical scavenging activity and ferrous ions-chelating ability are not the key factors that affect the antioxidant potential of AS. Therefore, we speculate that AS supply hydrogen or electrons and break free radical chains to achieve an antioxidant effect.

### Measurement of Effect Against H_2_O_2_-Induced Oxidative Stress

#### The Effect of H_2_O_2_ and AS on the Proliferation of RAW264.7 Cells

**Figure 2:**
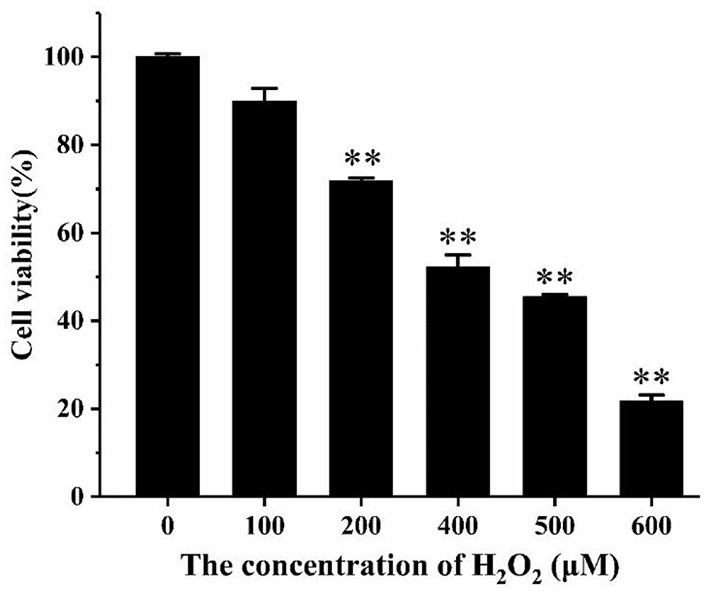
The effect of H_2_O_2_ on the viability of RAW264.7 cells. Cells were treated with different concentrations of H_2_O_2_ for 24 h. The results were presented as the mean ± SE of three independent experiments. **P < 0.01 compared with normal cell group (Supplementary Table 9).

CCK-8 analysis was used to determine the effect of different concentrations of H_2_O_2_ and AS on cell viability. It was apparent from Figure 2 that with the increasing concentration of hydrogen peroxide, cell viability decreased significantly. When the concentration of hydrogen peroxide was higher than 200 μM, the suppression of cell viability was extremely significant (*p* < 0.01). The cell viability was 52.29% at the concentration of 400 μM, indicating that IC_50_ of H_2_O_2_ was approximately 400 μM. Therefore, the H_2_O_2_ concentration of 400 μM was selected here for subsequent mechanism study.

**Figure 3:**
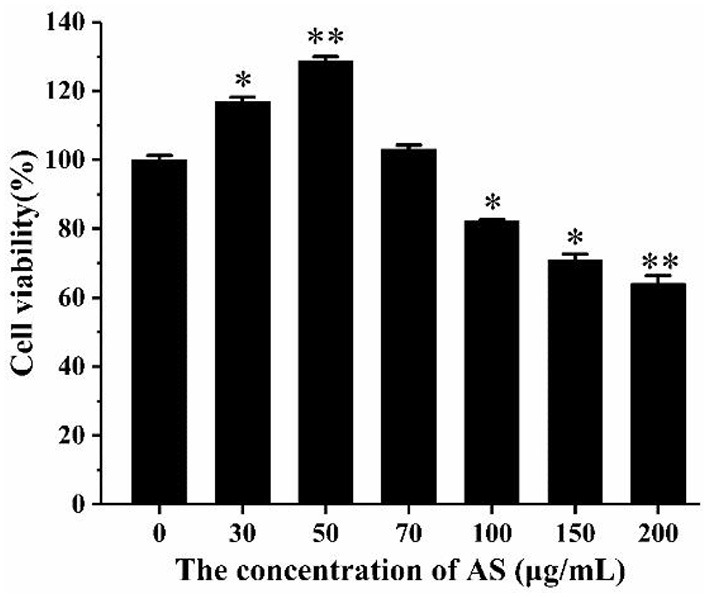
The effect of AS on the viability of RAW264.7 cells. Cells were treated with different concentrations of AS for 24 h. The results were presented as the mean ± SE of three independent experiments. *P < 0.05 and **P < 0.01 compared with the normal cell group (Supplementary Table 8).

As shown in Figure 3, when the cells were treated with AS at a concentration >100 μg/mL, a significant drop in cell viability was discovered (*p* < 0.05). This demonstrated that AS showed cytotoxic effect at 100 μg/mL and was not toxic to cells in the range of 30–70 μg/mL. Besides, the cell viability was significantly improved in the groups dealt with 30 μg/mL AS (*p* < 0.05), while the groups treated with 50 μg/mL AS had the highest cell viability (*p* < 0.01). We speculated AS may promote the proliferation of RAW264.7 cells within this concentration range. As a result, the low-, medium-, and high-dose of AS were selected as 30, 50, and 70 μg/mL, and taken the concentration as the recommended dose for subsequent experiments.

**Figure 4:**
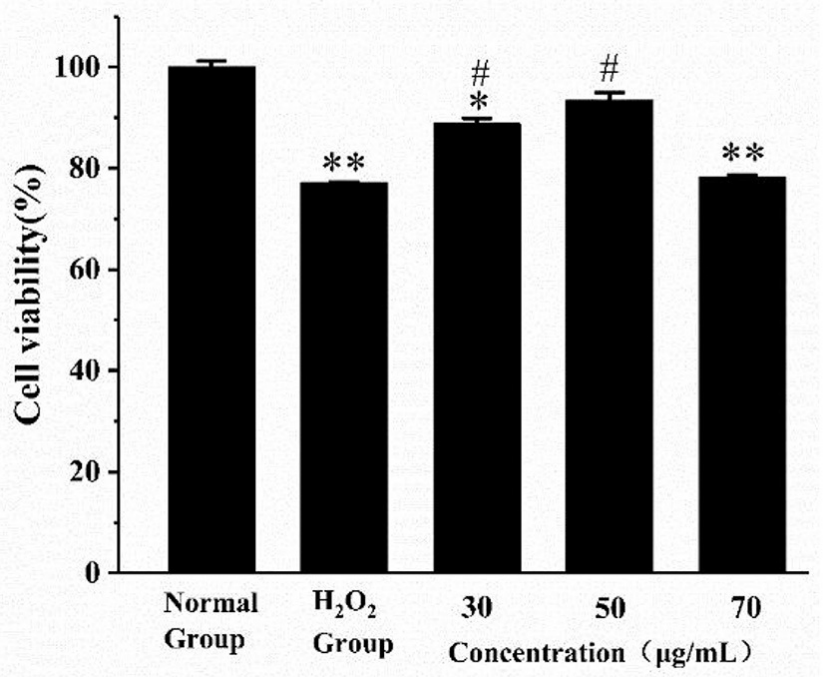
The effect of AS on the viability of RAW264.7 cells induced by H_2_O_2_. Cells were treated with different concentrations of AS for 20 h. The positive control group and AS-treated groups were then exposed to H_2_O_2_ (400 μM) for 4 h. The results were presented as the mean ± SE of three independent experiments. *P < 0.05 compared with the normal cell group, **P < 0.01 compared with the normal cell group. ^#^*P* < 0.05 compared with H_2_O_2_, ANOVA analyses (Supplementary Table 7).

As shown in Figure 4, the cell viability of the H_2_O_2_ group was significantly reduced compared with the normal group (*p* < 0.01). The cell viability was significantly improved in the low- and medium-dose groups (*p* < 0.05). When the AS concentration was 70 μg/mL, there was no significant difference in cell viability compared with the H_2_O_2_ group. The result of the H_2_O_2_ group revealed 400 μM H_2_O_2_ and 4 h incubation was sufficient to suppress the multiplication of RAW264.7 cells. What is more, low and medium doses of AS showed significant protective effect on the viability of H_2_O_2_-treated cells. According to previous antioxidant assay *in vitro*, AS was highly related to free radicals including hydroxyl radical scavenging effects, and this could further explain that AS showed improvement to the viability of RAW264.7 cells may benefit from its antioxidant.

#### Evaluation of Antioxidant Enzyme Activity and Lipid Peroxidation

**Figure 5:**
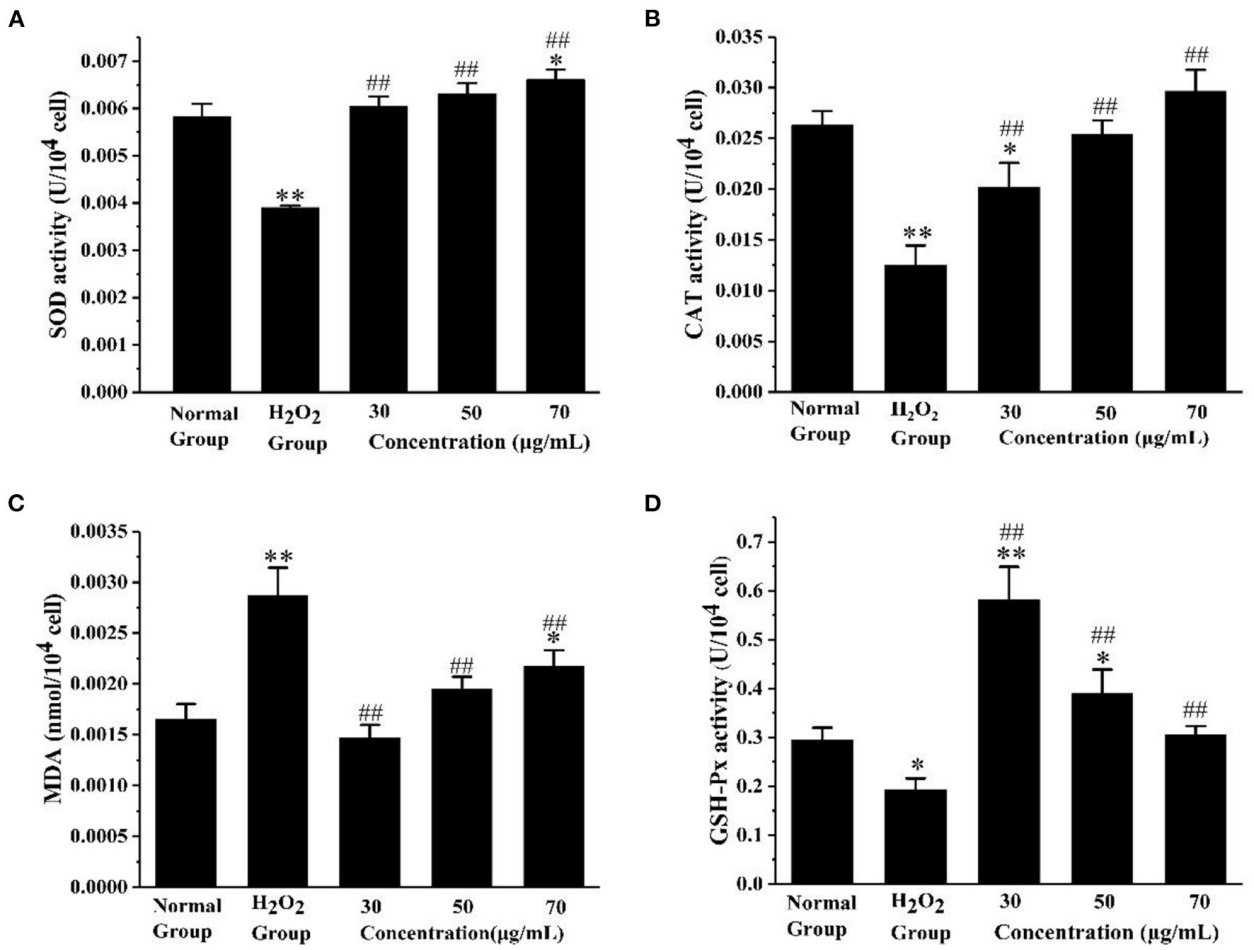
Evaluation of antioxidant enzyme activity and lipid peroxidation. **(A)** The effect of AS on the activity of SOD. **(B)** The effect of AS on the activity of CAT. **(C)** The effect of AS on the cellular concentration of MDA. **(D)** The effect of AS on the activity of GSH-Px. The results were expressed as the mean ± SE of three independent experiments. *P < 0.05 compared with the normal cell group, **P < 0.01 compared with the normal cell group. ^##^*P* < 0.01 compared with H_2_O_2_, ANOVA analyses (Supplementary Table 5).

In order to know more about the antioxidant effect of AS, we measured the antioxidant enzyme activity and lipid peroxidation degree of the cells treated with AS under oxidative stress. As shown in Figure 5A, SOD activity significantly increased in the three groups treated with AS compared with H_2_O_2_ group (*p* < 0.01). In addition, we found CAT activity in the AS-treated groups was significantly higher than that in the positive control group (*p* < 0.01) in Figure 5B. Both the medium-dose and high-dose groups recovered to levels indistinguishable from the negative control group (*p* > 0.05). As can be seen from Figure 5C, the MDA level of the H_2_O_2_ group was significantly higher than that of the negative control group after the cells were damaged by H_2_O_2_ (*p* < 0.01). After AS treatment, the MDA level of cells was significantly lower than that of the H_2_O_2_ group (*p* < 0.01). From Figure 5D we could see the GSH-Px activity in AS-treated groups was significantly up-regulated in comparison with the H_2_O_2_-treated group (*p* < 0.01). In addition, the activity of GSH-Px in the low-dose group was the highest among the AS-treated groups. Moreover, the MDA experiment showed that the oxidative damage level of the low-dose group is the lowest, consistent with this result.

These results indicated H_2_O_2_ caused oxidative damage to RAW264.7 cells, and the three doses of AS all had a certain protective effect on cells and effectively reduced the oxidative damage of macrophages. SOD catalyzes superoxide anions into H_2_O_2_ and O_2_, to achieve the purpose of scavenging free radicals. It plays a crucial role in the balance of oxygen utilization by the body (38). Both CAT and GSH-Px are important peroxidase enzymes that exist widely in the body. GSH-Px catalyzes glutathione (GSH) to form glutathione (oxidized) (GSSG), and that CAT collaborates with GSH-Px to reduce toxic hydrogen peroxide to non-toxic hydroxyl compounds (39, 40). MDA is one of the important products of membrane lipid oxidation, and its content reflects the level of free radical attack and indirectly indicates the damage degree of the cell membrane system (41). Through these findings, three doses of AS all alleviated the oxidative damage to a certain extent, decreased the MDA level, and maintained the oxidative balance of cells. Here, we confirm AS can increase the activities of SOD, CAT, and GSH-Px to reduce the damage of H_2_O_2_ to cells.

### Network Pharmacology Analysis

#### Related Gene Targets Database Construction

The 29 active components of AS were collected through the HERB database (Supplementary Table 3), and 26 active compounds were obtained by setting DL ≥ 0.18 in Swiss ADME (Table 2). According to the active components, we obtained 556 targets by using the Swiss Target Prediction database after removing the duplicates (Supplementary Table 1).

**Table 2:** Information of bioactive components of AS.

**Ingredient ID**	**CAS**	**Ingredient name**	**DL**
HBIN003737	1981-81-3	Hydroxyhopanone	0.55
HBIN004383	67392-96-5	Stigmast-4-en-3-one	0.55
HBIN007288	149-91-7	Gallic acid	0.56
HBIN008266	2034-74-4	3-Hydroxystigmast-5-en-7-one	0.55
HBIN012810	36450-02-9	(6beta,24R)-6-Hydroxystigmast-4-en-3-one	0.55
HBIN015955	559-70-6	Amyrin	0.55
HBIN018278	83-46-5	beta-Sitosterol	0.55
HBIN023517	514-07-8	Taraxerone	0.55
HBIN025629	169567	Erybidine	0.55
HBIN025688	5317205	Erythraline	0.55
HBIN025690	442220	Erythratidine	0.55
HBIN025796	305-01-1	Esculetin	0.55
HBIN029763	34427-61-7	Hydroxysitosterol	0.55
HBIN029818	487-58-1	Hypaphorine	0.55
HBIN029831	9065764	Hyperin	0.55
HBIN031753	520-18-3	Kaempferol	0.55
HBIN035817	69-65-8	Mannitol	0.55
HBIN041495	117-39-5	Quercetin	0.55
HBIN044152	474-58-8	Sitogluside	0.55
HBIN044849	113626-76-9	Stigmast-4-ene-3,6a-diol	0.55
HBIN044850	23670-94-2	Stigmast-4-ene-3,6-dione	0.55
HBIN044913	22149-69-5	Stigmastane-3,6-dione	0.55
HBIN046124	14167-59-0	Tetratriacontane	0.55
HBIN047613	77-52-1	Ursolic acid	0.85
HBIN047744	121-33-5	Vanillin	0.55
HBIN048051	14985	Vitamin E	0.55

#### Common Target Genes of Drugs and Diseases Database Construction

The 998 potential targets were found in Genecards database after using “anti-oxidant” as a keyword to search. The targets of the active components of AS were intersected with the antioxidant targets, and then we obtained 152 targets related to the antioxidant effect of AS.

#### Construction of Protein-Protein Interaction Network and Screening of Core Targets

**Figure 6:**
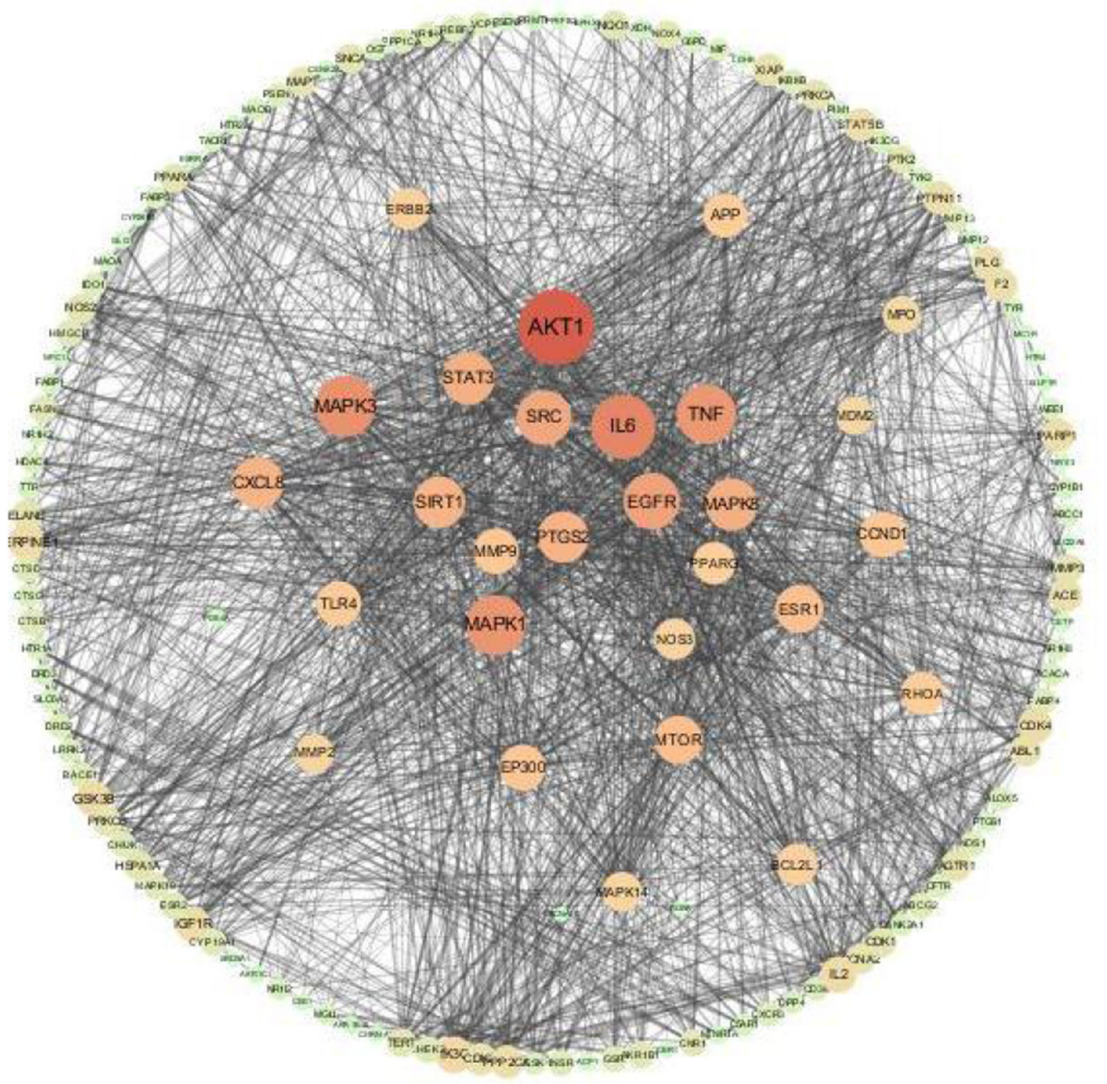
Antioxidant targets of AS interaction network.

The 152 intersection targets were imported into the String database to obtain the PPI relationship, and the PPI network was constructed on medium confidence interaction score (0.4) (Figure 6). The size and color of the nodes were adjusted according to the degree value. As the degree value increased, the nodes got bigger, and the color got darker (Supplementary Table 2). PPI network analysis results confirmed that the highest combined node score was 0.999 and the lowest combined score was 0.4 (Supplementary Table 11). The top 14 key target proteins were screened according to the degree value, and the results are shown in Table 3.

**Table 3:** Key targets of AS antioxidant PPI network.

**Number**	**Target**	**Degree**	**Number**	**Target**	**Degree**
1	Threonine kinase1 (AKT1)	106	2	Interleukin 6 (IL-6)	87
3	Mitogen-activated protein kinase 3 (MAPK3)	81	4	Mitogen-activated protein kinase 1 (MAPK1)	79
5	Tumor necrosis factor (TNF)	78	6	Epidermal growth factor receptor (EGFR)	73
7	SRC proto-oncogene (SRC)	70	8	Signal transducer and activator of transcription 3 (STAT3)	66
9	Mitogen-activated protein kinase 8 (MAPK8)	66	10	C-X-C motif chemokine ligand 8 (CXCL8)	64
11	Sirtuin-1 (SIRT1)	64	12	Prostaglandin-endoperoxide synthase 2 (PTGS2)	64
13	Mechanistic target of rapamycin kinase (MTOR)	59	14	Strogen receptor 1 (ESR1)	58

#### GO Analysis and KEGG Enrichment Analysis of Core Target Gene

**Figure 7:**
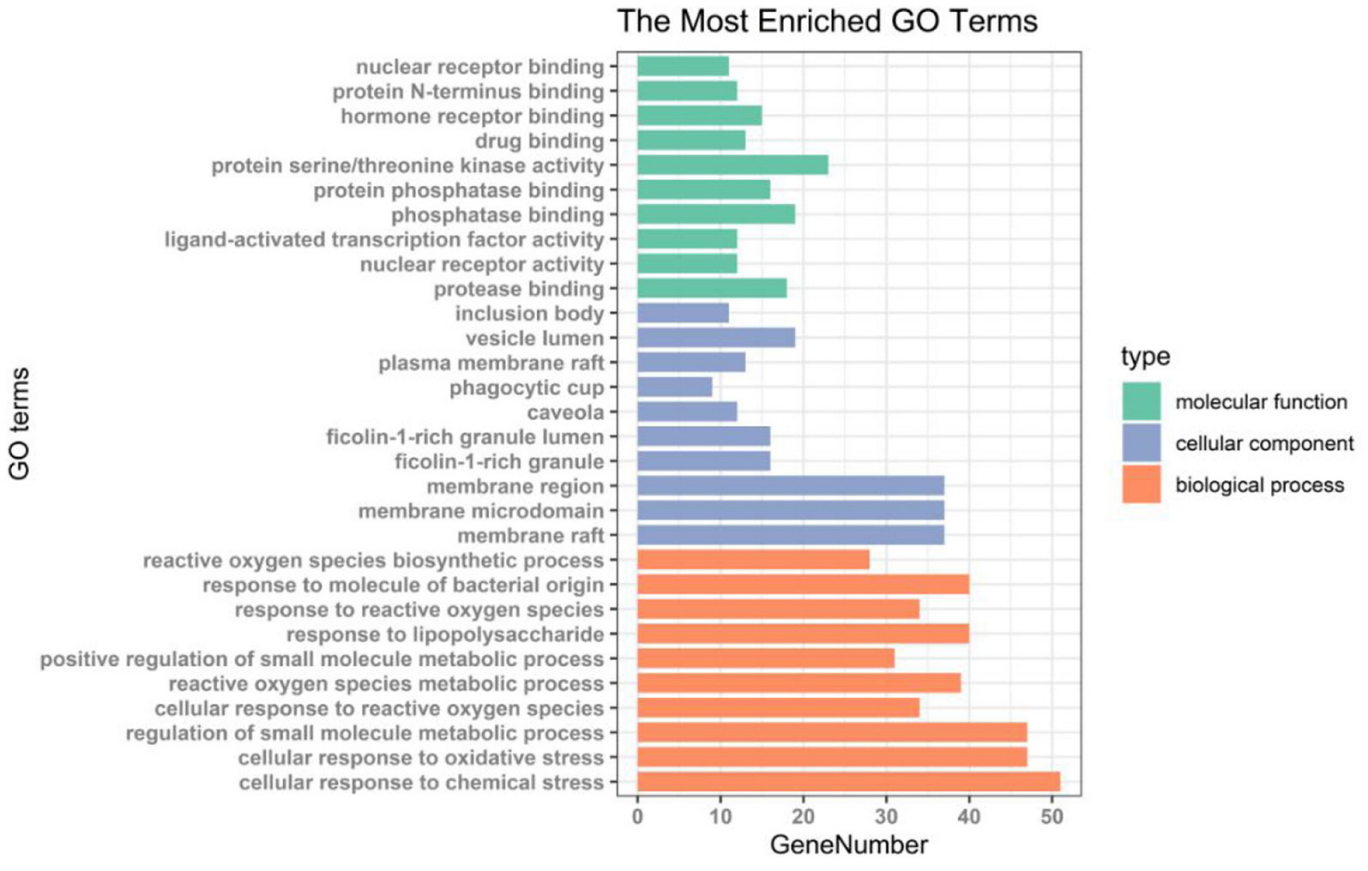
Histogram of GO enrichment analysis of antioxidant targets in AS.

We performed GO enrichment analysis and KEGG pathway annotation analysis on 152 intersection targets. GO analysis resulted in 2,837 GO entries (*P* < 0. 05), 2,544 items for biological process (BP), 114 items for cell component (CC), and 179 items for molecular function (MF) (Supplementary Data Sheet 1). Among these categories, most targets were enriched in the biological process. Within the biological process category, reactive oxygen species biosynthetic process and response to molecules of bacterial origin were the most dominant subcategories. About the molecular function category, the most targets were assigned to nuclear receptor binding, protein N-terminus binding, hormone receptor binding, and drug binding. As for the cellular components category, the four most abundant sub-categories were inclusion body, vesicle lumen, plasma membrane raft, and phagocytic cup (Figure 7).

**Figure 8:**
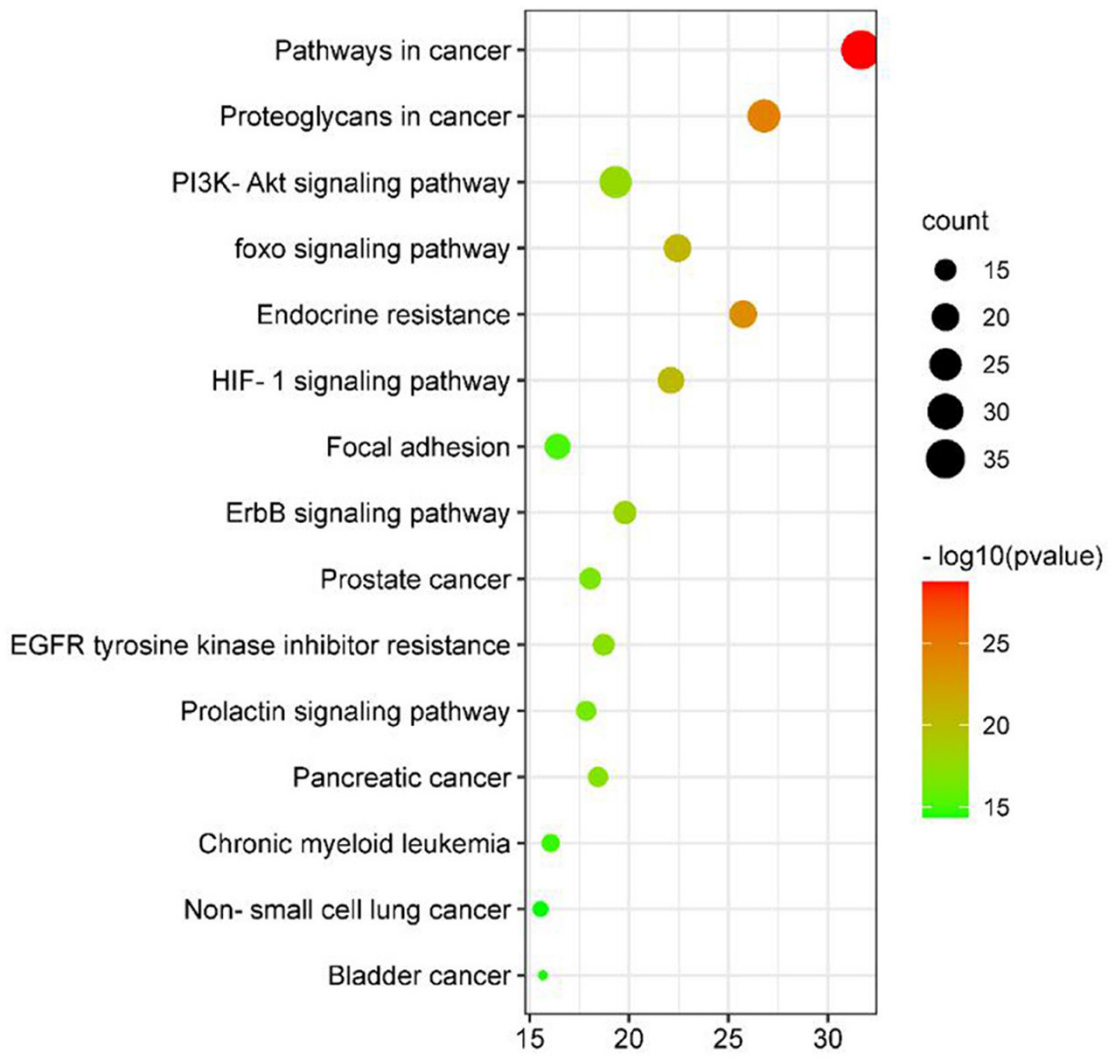
Bubble chart of KEGG pathway enrichment analysis of antioxidant targets in AS.

We used KEGG analysis to analyze the regulatory pathways of the targets and obtained 212 signaling pathways (*p* < 0.05) (Supplementary Table 6). Figure 8 shows the top 15 potential signal pathways of the targets. The KEGG pathways in which most targets were enriched were pathways in cancer, proteoglycans in cancer, PI3K-AKT signaling pathway, and FoxO signaling pathway. Moreover, the oxidative stress related pathways were PI3K-AKT signaling pathway and FoxO signaling pathway. A total of 20 target genes including AKT1, SIRT1, and MAPK1 were involved in the FoxO pathway, and 25 target genes including AKT1, MTOR, and MAPK1 were involved in the PI3K/AKT pathway. We selected the top 4 antioxidant effect related genes (MTOR, AKT1, SIRT1, and MAPK1) belonging to these pathways to further confirmation under qPCR experiment.

The forkhead box O (FoxO) family of transcription factors regulates the expression of genes in cellular physiological events including apoptosis, cell-cycle control, glucose metabolism, oxidative stress resistance, and longevity (42). A central regulatory mechanism of FoxO proteins is phosphorylation by the serine-threonine kinase AKT/protein kinase B (AKT/PKB), downstream of phosphatidylinositol 3-kinase (PI3K), in response to insulin or several growth factors (43). Studies have shown that FoxO1 can reduce oxidative stress injury by regulating downstream target genes, such as Mn-superoxide dismutase (Mn-SOD) and catalase (CAT), to remove excess ROS (44). Phosphatidylinositol-3-kinase/proteinkinase B (PI3K/ AKT) signaling pathway is an important pathway for intracellular transduction of membrane receptor signals. It regulates cardiovascular function through various mechanisms such as vascular endothelial cell migration, angiogenesis, and energy metabolism, and is closely related to oxidative stress and inflammatory response (45). Previous studies have confirmed that some important signal transduction pathways, such as PI3K-AKT signaling pathway, deal with the oxidative damage to cells by participating in ROS activation of Nrf2 (46). Therefore, we supposed AS exerts antioxidant effect mainly through affecting FoxO and PI3K/AKT signaling pathway.

#### The Expression Level of AS-Antioxidant-Related Genes

**Figure 9:**
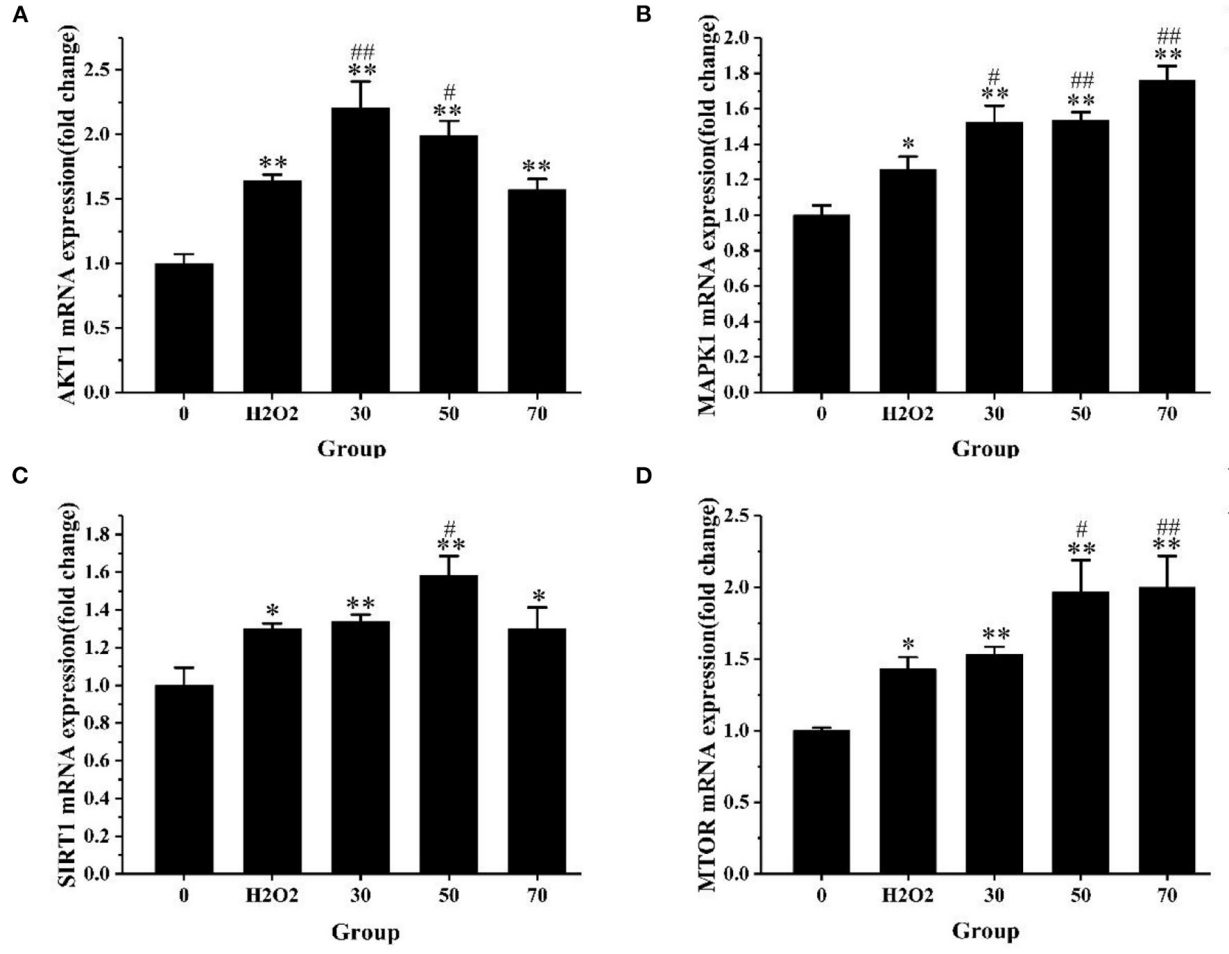
The expression level of core genes. **(A)** The expression level of AKT1 mRNA. **(B)** The expression level of MAPK1 mRNA. **(C)** The expression level of SIRT1 mRNA. **(D)** The expression level of MTOR mRNA. The results were expressed as the mean ± SE of three independent experiments. *P < 0.05 vs. 0, **P < 0.01 vs. 0. ^#^*P* < 0.05 vs. H_2_O_2_, ^##^*P* < 0.01 vs. H_2_O_2_, ANOVA analyses (Supplementary Table 10).

To explore the molecular mechanism of AS in the treatment of oxidative stress, we selected AKT1, MTOR, MAPK1, and SIRT1 to verify the expression level changes by qRT-PCR. These genes were selected based on the result of KEGG analysis. After 4 h of H_2_O_2_ treatment, we detected the expression levels of these four target genes. As exhibited in Figure 9, the expression levels of AKT1 in the low-dose and medium-dose groups were significantly up-regulated compared with the positive control group (*p* < 0.05). MAPK1 gene expression levels increased significantly in all AS treatment groups (*p* < 0.05), the expression levels of MTOR in the medium- and high-dose groups were significantly up-regulated (*p* < 0.05). SIRT1 expression was significantly up-regulated in the medium-dose group (*p* < 0.05).

Sirtuin-1 (SIRT1) is a conserved, nicotinamide adenine dinucleotide (NAD+)-dependent III histone deacetylase (47). Immense amounts of studies have shown that SIRT1 plays an important role in oxidative stress injury by regulating various target genes and proteins such as NF-κB, FoxO1, P53, and Nrf2 (48–51). SIRT1 can activate FoxO1 through deacetylation and alleviate oxidative stress injury caused by H_2_O_2_ (49). AKT1 is a downstream molecule of SIRT1, which has a significant influence on the regulation of cell proliferation, cell survival, and protein synthesis (52). Zhai et al. (53) have proved that overexpressed SIRT1 increases the phosphorylation levels of PI3K and AKT, thereby inhibiting the apoptosis of cardiomyocytes induced by high glucose and reducing its oxidative stress response. MTOR is an important downstream target of AKT and takes part in the expression and transcription of related proteins and genes, thus affecting biological activities such as inflammation, oxidative stress, apoptosis, and so on (54, 55). The over-expressed SIRT1 activates PI3K through tyrosine kinase receptor, then the activated PI3K promotes the phosphorylation of AKT, activates MTOR, and inhibits oxidative stress and inflammation (56). These studies have revealed that over-expressed SIRT1 activates MTOR and AKT1 and reduces oxidative stress injury. This is consistent with the fact that AS can up-regulate the expression levels of SIRT1, MTOR, and AKT1 in this experiment. MAPK signaling pathway is an important pathway that controls many basic cellular processes such as cell proliferation, oxidative stress, survival, and apoptosis (57). Therefore, MAPK1 may play an important role in the regulation of oxidative stress in cells.

**Figure 10:**
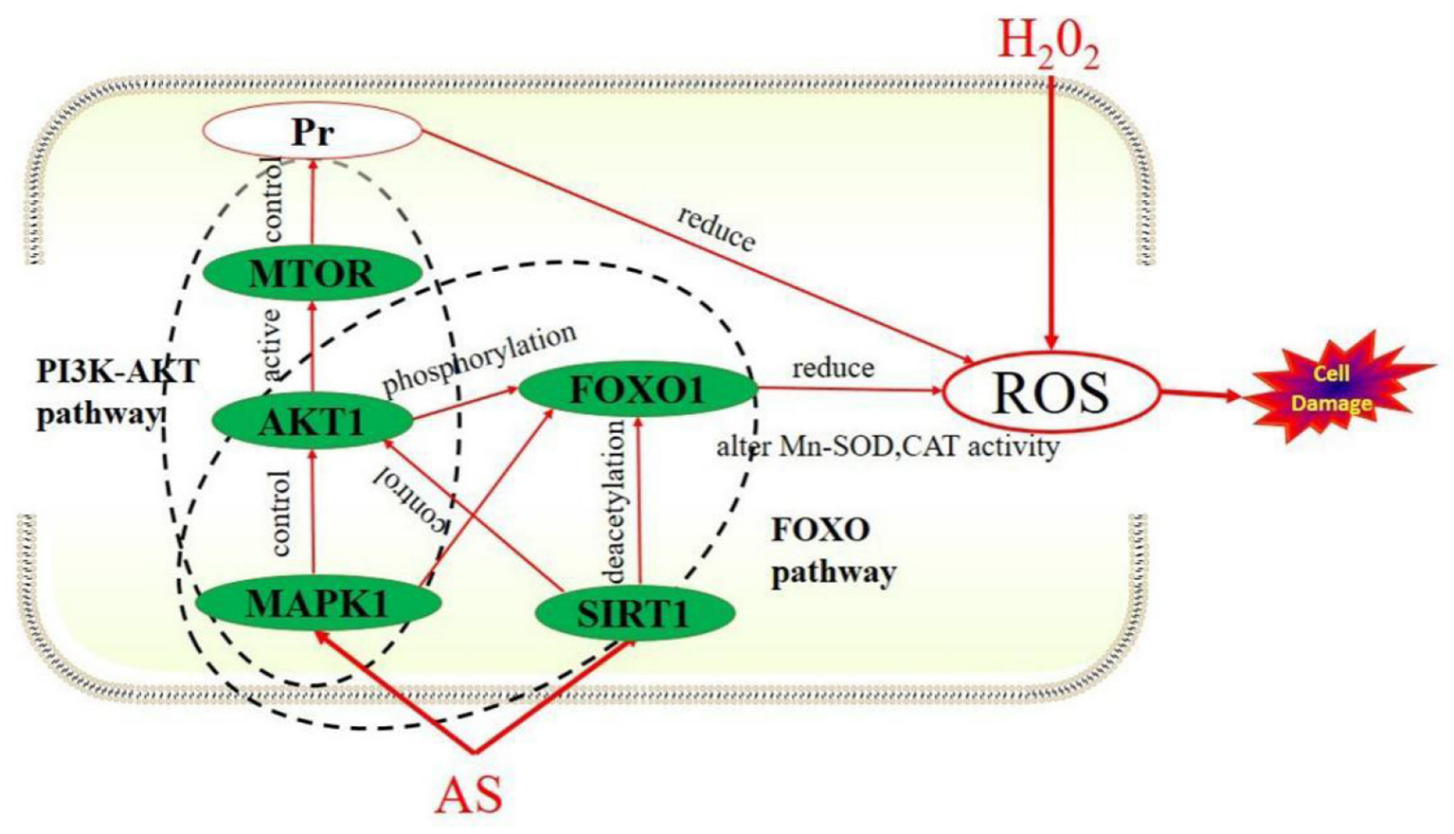
How AS exerts antioxidant effects.

The results showed the expression levels of AKT1, MAPK1, SIRT1, and MTOR in the model group were significantly up-regulated compared with the normal group (*p* < 0.05). After AS treatment, the expression levels of these four target genes were increased again compared with the H_2_O_2_ group. We speculated that after a short period of oxidative stress, the cell's antioxidant mechanism will be activated by excessive ROS, that is alleviating the oxidative damage of cells through self-regulation and compensatory up-regulate these four genes. In this study, PI3K-AKT and FoxO signaling pathway were the key signaling pathways obtained from KEGG pathway enrichment analysis. What is more, AKT1, SIRT1, and MAPK1 could regulate the FoxO signaling pathway, while AKT1, MTOR, and MAPK1 can regulate the PI3K/AKT signaling pathway. The mechanism of how AS exerts its antioxidant effect we predicted was shown in Figure 10.

These results suggested that FoxO and PI3K-AKT signaling pathways might be the key pathways for AS to exert antioxidant effects. This prediction result is consistent with the fact that AS can up-regulate the expression of these four target genes. In conclusion, AS mitigates oxidative damage that may be attributed to it regulating FoxO and PI3K-AKT signaling pathways by up-regulating AKT1, SIRT1, MTOR, and MAPK1.

## Conclusions

The present study investigated the *in vitro* and intracellular antioxidant activity of AS, and the potential antioxidant mechanism based on network pharmacology. In *in vitro* studies, AS revealed the strong reducing power and DPPH scavenging effect, but hydroxyl radical scavenging activity and ferrous ions-chelating ability were not strong. The intracellular studies of RAW264.7 cells presented pretreatment with AS significantly improved the antioxidant status of cells. AS showed significant protective effect on the viability of H_2_O_2_-treated cells, increased the activities of SOD, CAT, and GSH-Px, and decreased the MDA level. We used network pharmacology analysis to select core targets (MTOR, AKT1, SIRT1, and MAPK1) belonging to FoxO and PI3K/AKT signaling pathway to further confirmation. AS preconditioning could significantly up-regulate the expression level of AKT1, SIRT1, MAPK1, and MTOR in model cells, and the effect was related to the regulation of FoxO and PI3K/AKT signaling pathway. It can be inferred that the accuracy of this network pharmacology study is high and worth further study.

## Data Availability Statement

The original contributions presented in the study are included in the article/Supplementary Material, further inquiries can be directed to the corresponding author.

## Author Contributions

Y-nM: writing-original draft, visualization, data curation, formal analysis, and investigation. FC: conceptualization, methodology, software, and writing-review and editing. ZY: writing-review and editing, investigation, and resources. X-fS: writing-review and editing and conceptualization. J-pL, H-jZ, AW, and C-fL: funding acquisition. R-fS and B-cH: supervision and funding acquisition. X-hW: supervision. YL: funding acquisition, resources, writing-review and editing, and project administration. All authors contributed to the article and approved the submitted version.

## Funding

This work was supported by Agricultural Science and Technology Innovation Program (No. 25-LZIHPS-03), Lanzhou Science and Technology Planning Project (No. 2018-1-114), and Xinjiang Uygur Autonomous Region “Tianchi Doctoral Project” (No. E1954101).

## Conflict of Interest

The authors declare that the research was conducted in the absence of any commercial or financial relationships that could be construed as a potential conflict of interest.

## Publisher's Note

All claims expressed in this article are solely those of the authors and do not necessarily represent those of their affiliated organizations, or those of the publisher, the editors and the reviewers. Any product that may be evaluated in this article, or claim that may be made by its manufacturer, is not guaranteed or endorsed by the publisher.
